# Supplementary material for: Predicting Multimorbidity Using Saudi Health Indicators (Sharik) Nationwide Data: Statistical and Machine Learning Approach
Source: Healthcare (Basel). 2023 Jul 31;11(15):2176. doi: 10.3390/healthcare11152176 (PMC10418949; doi:10.3390/healthcare11152176)
Supplement: Supplementary file 1 [file healthcare-11-02176-s001.zip › healthcare-2474254-supplementary.pdf]

**Supplementary Table S1: Variables coding**

| Variable | Coding | Label |
|---|---|---|
| **Gender** | 0 | Male |
| | 1 | Female |
| Vegetable intake | 0 | never |
| | 1 | 1 day |
| | 7 | 7 days |
| Fruit intake | 0 | never |
| | 1 | 1 day |
| | 7 | 7 days |
| intense physical activity | 0 | never |
| | 1 | 1 day |
| | 7 | 7 days |
| moderate physical activity | 0 | never |
| | 1 | 1 day |
| | 7 | 7 days |
| Diabetes | 0 | no |
| | 1 | yes |
| High cholesterol | 0 | no |
| | 1 | yes |
| Hypertesnion | 0 | no |
| | 1 | yes |
| Genetic disorder | 0 | no |
| | 1 | yes |
| Stroke | 0 | no |
| | 1 | yes |
| Cancer | 0 | no |
| | 1 | yes |
| Chronic lung disease | 0 | no |
| | 1 | yes |
| Asthma | 0 | no |
| | 1 | yes |
| Obese | 0 | no |
| | 1 | yes |
| Smoker | 0 | no |
| | 1 | yes |
| Multimorbidity | 0 | no |
| | 1 | yes |
